# *In Vitro* Drug Sensitivity Tests to Predict Molecular Target Drug Responses in Surgically Resected Lung Cancer

**DOI:** 10.1371/journal.pone.0152665

**Published:** 2016-04-12

**Authors:** Ryohei Miyazaki, Takashi Anayama, Kentaro Hirohashi, Hironobu Okada, Motohiko Kume, Kazumasa Orihashi

**Affiliations:** Division of Thoracic Surgery, Department of Surgery II, Kochi Medical School, Kochi University, Nankoku, Kochi, Japan; University of Colorado, School of Medicine, UNITED STATES

## Abstract

**Background:**

Epidermal growth factor receptor-tyrosine kinase inhibitors (EGFR-TKIs) and anaplastic lymphoma kinase (ALK) inhibitors have dramatically changed the strategy of medical treatment of lung cancer. Patients should be screened for the presence of the EGFR mutation or echinoderm microtubule-associated protein-like 4 *(EML4*)-*ALK* fusion gene prior to chemotherapy to predict their clinical response. The succinate dehydrogenase inhibition (SDI) test and collagen gel droplet embedded culture drug sensitivity test (CD-DST) are established *in vitro* drug sensitivity tests, which may predict the sensitivity of patients to cytotoxic anticancer drugs. We applied *in vitro* drug sensitivity tests for cyclopedic prediction of clinical responses to different molecular targeting drugs.

**Methods:**

The growth inhibitory effects of erlotinib and crizotinib were confirmed for lung cancer cell lines using SDI and CD-DST. The sensitivity of 35 cases of surgically resected lung cancer to erlotinib was examined using SDI or CD-DST, and compared with EGFR mutation status.

**Results:**

HCC827 (Exon19: E746-A750 del) and H3122 (*EML4-ALK*) cells were inhibited by lower concentrations of erlotinib and crizotinib, respectively than A549, H460, and H1975 (L858R+T790M) cells were. The viability of the surgically resected lung cancer was 60.0 ± 9.8 and 86.8 ± 13.9% in EGFR-mutants vs. wild types in the SDI (p = 0.0003). The cell viability was 33.5 ± 21.2 and 79.0 ± 18.6% in EGFR mutants vs. wild-type cases (p = 0.026) in CD-DST.

**Conclusions:**

*In vitro* drug sensitivity evaluated by either SDI or CD-DST correlated with EGFR gene status. Therefore, SDI and CD-DST may be useful predictors of potential clinical responses to the molecular anticancer drugs, cyclopedically.

## Introduction

Lung cancer is the leading cause of cancer-related mortality in many developed countries while adenocarcinoma represents 70% of the cases of non-small cell lung cancer (NSCLC). In Japanese patients, approximately 50 and 5% of adenocarcinomas have a mutation in the epidermal growth factor receptor (EGFR) [1] and echinoderm microtubule-associated protein-like 4-anaplastic lymphoma kinase (*EML4-ALK*) fusion gene, respectively. Molecular target anticancer drugs such as EGFR-tyrosine kinase inhibitors (TKIs) and ALK inhibitors have dramatically changed the strategy of clinical treatment of cancer.

Most EGFR mutation-positive lung cancers are sensitive to EGFR-TKIs, which contribute to extending the progression-free survival (PFS) and overall survival (OS) of patients [1–4]. However, EGFR mutation-positive cases do not always exhibit good clinical responses to EGFR TKI therapy. Approximately 30% of EGFR mutation cases are resistant to EGFR-TKIs [3, 4]. The mutation of the Kirsten rat sarcoma viral oncogene homolog (K-RAS) and serine/threonine-protein kinase B-Raf (B-RAF) or second mutations such as T790M are known to correlate with resistance to EGFR-TKIs.[5] On the other hand, about 10% of the EGFR wild-type cases exhibit clinical responses to EGFR-TKIs [4]. Furthermore, EGFR-TKIs are also effective in cases of squamous cell carcinoma that commonly do not show the EGFR gene mutation [6]. Therefore, there are some populations of EGFR-TKI responders who are not screened for EGFR mutations.

*EML4-ALK*-positive lung cancer is a primary malignant lung tumor consisting of cells that with a characteristic abnormal configuration of the DNA where the *EML4* gene is fused to the anaplastic lymphoma kinase (*ALK*) gene. *ALK*-inhibitors (crizotinib or alectinib) are currently available for clinical use. It is common to confirm the presence of *EML4-ALK* using a fluorescence in situ hybridization (FISH) analysis but high-sensitivity immunostaining has been alternatively utilized for the screening of *EML4-ALK*. However, FISH and immunostaining do not necessarily relate to the clinical response rate to *ALK* inhibitors in practice [7–9]. The development of molecular target drugs for new driver mutations should be required to include investigate of the responsible gene mutation. There is currently no established method to comprehensively predict the effect of molecular targeting agents that act at different points of the various signaling pathways.

There are in vitro anticancer drug sensitivity tests such as the succinate dehydrogenase inhibition (SDI) test, histoculture drug response assay (HDRA) method, and collagen gel droplet embedded culture drug sensitivity test (CD-DST). Interestingly, the order-maid chemotherapy with anticancer drugs, which were predicted as effective, actually exhibited higher clinical responses than the conventional chemotherapy did [10–12]. Furthermore, there is a report suggesting that the in vitro drug sensitivity test may be useful in predicting the effect of adjuvant chemotherapy in NSCLC.[13] However, there has been no previous report on the clinical application of in vitro drug sensitivity tests for the prediction of the potential effects of EGFR-TKIs or *ALK* inhibitors in surgically resected fresh lung cancer tissue specimens. The purpose of the current study was to develop an in vitro culture-based drug sensitivity test to predict the sensitivity of surgically resected lung cancer to multiple molecular target drugs.

## Materials and Methods

### A. Verification of inhibitory effect of molecular target drugs in lung cancer cell line

The optimal doses of erlotinib and crizotinib (Funakoshi Co. Ltd, Tokyo, Japan) used in both the 3-(4,5-dimethylthiazol-2-yl)-5-(3-carboxymethoxyphenyl)-2-(4-sulfophenyl)-2H-tetrazolium (MTS) assay and CD-DST were titrated using the following immortalized human lung cancer cell lines: HCC827 (adenocarcinoma, EGFR mutation on Exon19, E746-A750 deletion), H1975 (adenocarcinoma, EGFR mutation, Exon 21 L858R+T790M), H3122 (adenocarcinoma, *EML4-ALK* fusion gene), A549 (adenocarcinoma), and H460 (large cell carcinoma). A549, H460, HCC827 and H1975 were obtained from American Type Culture Collection (Manassas, Virginia USA). H3122 was kindly provided by Dr. Pasi A. Jänne of Harvard Medical School (Boston, MA, USA).

#### A-(1) MTS assay in human lung cancer cell lines

The cancer cells were transferred into 96-well flat-bottom culture plates at a density of 4.0 × 10^4^–6.0 × 10^4^ cells/well with increasing concentrations of erlotinib (0.002, 0.02, 0.2, 2.0, and 20 μM) and crizotinib (0.006, 0.06, 0.6, and 3 μM), and incubated at 37°C for 72 h exposed to 5% CO_2_. After adding 20 μL of Cell Titer (Promega Corporation, Madison, WI, USA) to the culture medium, the cancer cells were incubated for 90 min and the absorbance was measured at 490 nm.

#### A-(2) Immunoblot analysis

Immunoblot analysis was conducted as previously described [14, 15] Cells were washed with PBS, harvested, and lysed in RIPA buffer (Nacalai Tesque inc., Kyoto, Japan) with Phosphatase Inhibitor Cocktail (Nacalai Tesque inc., Kyoto, Japan). For Western blot analysis, 30μg of total extracts were suspended in 20μl Sample Buffer Solution with 2-ME for SDS-PAGE (Nacalai Tesque inc., Kyoto, Japan), were electrophoretically resolved on denaturing polyacrylamide gels, transferd to nitrocellulose. The membranes were blocked with Blocking One or Blocking One-P (Nacalai Tesque inc., Kyoto, Japan), and probed with rabbit monoclonal antibodies to phosphorylated human ALK (Y1604), to ALK, the phosphorylated EGF receptor (Y1068), and to the EGF receptor (Cell Signaling Technology, Beverly, MA, USA). Then, the membranes were incubated in anti-rabbit secondary antibody (Santa Cruz Biotechnology, Dallas, Texas, USA). Each band was scanned by LAS -4000.mini(FUJIFILM, Tokyo, Japan).

#### A-(3) CD-DST in human lung cancer cell lines

The CD-DST was performed according to a previously reported method [16]. Briefly, the cancer cell lines were treated with a cell dispersion enzyme solution (EZ, Nitta Gelatin Inc., Osaka, Japan) for 2 h. Then, only viable cells that adhered to the collagen gel were collected and suspended in the reconstructed type I collagen solution (Cellmatrix Type CD, Nitta Gelatin Inc., Osaka, Japan) at a final density of 1 × 10^5^ cells/mL. Three drops of the collagen-cell mixture (30 μL/drop) were placed in each well of a 6-well multiplate in a 60-mm dish, and allowed to gel at 37°C in a CO2 incubator for 1 h. The final concentration was about 3 × 10^3^ cells/collagen gel droplet. The culture medium was overlaid in each well and the plate was incubated in a CO_2_ incubator at 37°C overnight. Three collagen droplets were placed at the bottom of 6-well plates to enable culturing in a three dimensional (3-D) environment, and were incubated for 7 days in serum-free medium in the presence of the same concentration ranges of erlotinib and crizotinib used in the MTS assay. Then, the cells were fixed with 10% buffered-formalin (Nacalai Tesque inc., Kyoto, Japan), and stained with Neutral Red (Kurabo, Osaka, Japan). Images of the fixed cells were captured using microscope photography. The images including both cancer cells (deeply stained) and fibroblasts (lightly stained) stained with neutral red were acquired, Fibroblasts were selected and eliminated as the uniformed spindle cells with small-sized nuclei, then the number of viable cancer cells was quantified using a dedicated software (Primege, version 1.01, Nitta Gelatin Inc., Osaka, Japan) [17, 18]. The viability of erlotinib or crizotinib-treated cells was compared with that of the untreated control cells.

### B. Clinical study using surgically resected fresh lung cancer tissue specimens

This study was approved by the ethical committee of the Kochi Medical School Hospital (Ethics Review Approval Number: ERB-100866), and was conducted from June 2013 to August 2014. The all participants provided their written informed consent to participate in this study according to the consent procedure. Thirty-five patients with surgically resectable NSCLC (SDI and CD-DST, n = 23 and 12, respectively) were enrolled in the clinical study. After surgical resection, a part of fresh cancer tissue specimens, checked tumor cell by touch smear cytology, were obtained for the in vitro drug sensitivity tests as described in the following sections.

#### B-(1) SDI prediction of the sensitivity to erlotinib for clinical lung cancer tissue specimen

The SDI is the protocol based on MTS assay for the bulk fresh tissue specimen including both cancer cells and non-cancer cells [19, 20]. Briefly, the fresh surgical specimens (10 milliliter cubed: 1mL) were minced into a paste, treated with a cell dispersion enzyme at 37°C for 2–3 h, centrifuged, and then the supernatant was removed. Viable lung cancer cells were then transferred into 96-well plates at a density of 1.0 × 10^6^ cells/well and incubated in Dulbecco’s modified Eagle’s medium (DMEM) containing fetal bovine serum (FBS) at 37°C with 5% CO_2_ for 72 h. The cells were exposed to the same concentration range of erlotinib used in the MTS assay. Then, 20 μL of cell titer was added to each well, and the plates were incubated for 150 min followed by measurement of the absorbance at 490 nm to quantify the cell viability.

#### B-(2) CD-DST prediction of the sensitivity to erlotinib for clinical lung cancer tissue specimen

Fresh tissue specimens (3 milliliter cubed: 27μL) obtained from the surgically excised lung cancer tissue was minced finely using a scalpel and digested in a cell dispersion enzyme solution (EZ, Nitta Gelatin Inc., Osaka, Japan) for 2 h. The dispersed cells were washed twice, collected by centrifugation at 2400 rpm for 3 min, filtered through an 80-μm nylon mesh to obtain more than 1.0 × 10^5^ cells, which were incubated in a collagen gel coated flask (CG-flask, Nitta Gelatin Inc., Osaka, Japan) in a CO_2_ incubator at 37°C for 24 h. The recovered cells were then enclosed in a collagen droplet to be cultured in a 3D environment. Viable lung cancer cells were then incubated with 0.2 μM of erlotinib (optimal dose was determined in A-(2)) for 7 days. Prepared culture media (PCM) 2 was used in the standard protocol for CD-DST. However, significant findings were not obtained using the previous test method [21] and, therefore, PCM4 (Kurabo, Oosaka, Japan), a growth factor-reduced culture medium was used in the current study.

#### B-(3) *EGFR* gene mutation analysis

We screened for EGFR mutation using the peptide nucleic acid-locked nucleic acid polymerase chain reaction (PNA-LNA PCR) clamp method [22, 23]. The detailed gene mutation status of each sample was confirmed using the direct sequencing method. EGFR mutations in the extracted DNA were examined using the PCR-based direct sequencing for exons 19 and 21. Sequencing was carried out using the Applied Biosystems PRISM dye terminator cycle sequencing method (Perkin-Elmer Corp., Foster City, CA, USA) with an ABI PRISM 3100 Genetic Analyzer (Applied Biosystems, Foster City, CA, USA).

### C. Statistical analysis

We described the dose-response curve of the molecular targeting drugs for lung cancer cell lines. The correlation between the somatic gene mutations of *EGFR* in the cancer cells and drug sensitivity to erlotinib were compared. The patients were divided into two groups: wild-type and mutant EGFR groups. The results of the drug sensitivity test of both groups were statistically compared using the Mann-Whitney’s U-test. We refered to statistically significant as p<0.05. The ideal cut off value of cell viability designated to predict the cancer cells as sensitive to elrotinib was determined using a receiver operating characteristic (ROC) curve. Dose-response and ROC curves were constructed using the GraphPad Prism version 6.0 for Windows (GraphPad Software, San Diego, CA, USA).

## Results

### A. Inhibitory effect of molecular target drugs in lung cancer cell line

#### A-(1) MTS assay in lung cancer cell lines

**Fig 1:**
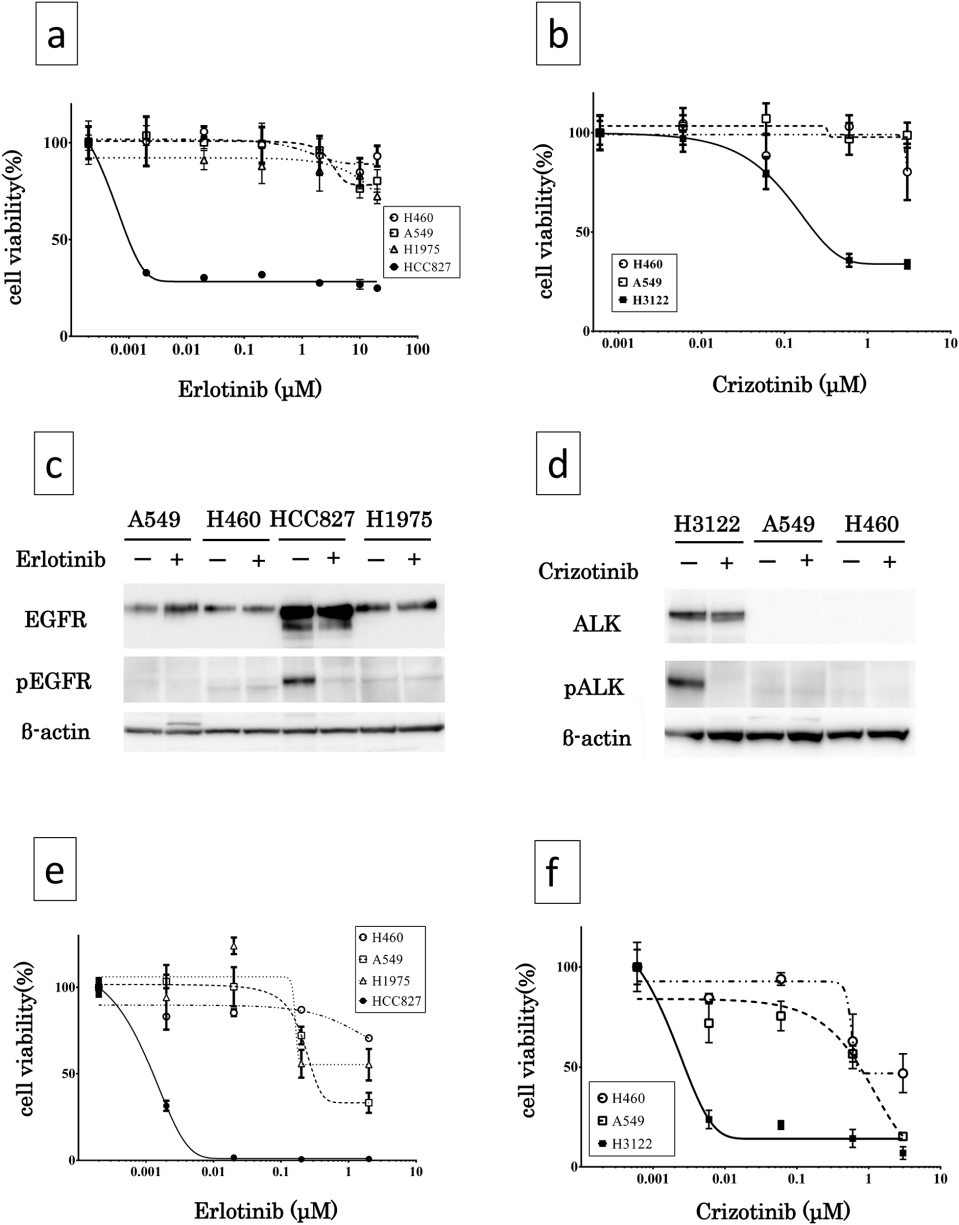
Dose dependent growth inhibition of lung cancer cell-lines by erlotinib and crizotinib. (a) Exposure to erlotinib in MTS assay: HCC827 (EGFR exon 19 deletion) exhibited significant growth inhibition while A549 and H460 (wild EGFR) or H1975 (EGFR T790M resistance second mutation) were not sensitive to elrotinib. (b) Exposure to crizotinib in MTS assay: H3122 (*EML4-ALK* fusion) exhibited significant growth inhibition, while other cancer cells without a fusion gene were not sensitive to crizotinib. (c) Expression of EGFR protein and inhibition of phosphorylation by erlotinib in NSCLC cell lines in western blot analysis: Erlotinib inhibited phosphorylation of EGFR (pEGFR) in HCC827, but did not inhibit it in H1975. (d) Expression of ALK and inhibition of phosphorylation by crizotinib in NSCLC cell lines in western blot analysis: H3122 expressed ALK and crizotinib inhibited phosphorylation of ALK(pALK). (e) Exposure to erlotinib in CD-DST. (f) Exposure to crizotinib in CD-DST. Compared with MTS assay, CD-DST required lower doses of erlotinib to exhibit significant difference in growth between cell lines. *EGFR*, epidermal growth factor receptor gene; *EML4-ALK*, echinoderm microtubule-associated protein-like 4-anaplastic lymphoma kinase gene.

The viability of the lung cancer cell lines following exposure to 2.0 μM of erlotinib for 3 days was 98.9 ± 9.3, 99.4 ± 10.7, 85.7 ± 10.7, and 31.9 ± 1.6 (%) for H460, A549, H1975, and HCC827, respectively. The growth of the HCC827 (with *EGFR* mutation, del E746_A750) but not that of the other cell lines without the mutation or with the 2^nd^ resistant mutation (T790M) cell line was significantly inhibited following exposure to erlotinib (p = 0.030, Fig 1-a). Similarly, the viability of the cell lines following exposure to 0.60 μM of crizotinib for 3 days was 103.2 ± 5.67, 96.9 ± 8.05, and 35.8 ± 3.24% for H460, A549, and H3122, respectively. H3122 (with *EML4-ALK* fusion gene) exhibited significant growth inhibition following exposure to crizotinib (Fig 1-b).

#### A-(2) Immunoblotting analysis

We examined EGFR and ALK expression in the tumor cells and the effect of erlotinib and crizotinib on the phosphorylation of EGFR and ALK by Western blotting. All cell lines expressed EGFR. The EGFR phosphorylation was suppressed by erlotinib in HCC827. On the other hand, erlotinib did not suppress the phosphorylation of EGFR in H1975 cells (Fig 1-c). H3122 cells expressed ALK, and the ALK phosphorylation was suppressed by crizotinib (Fig 1-d).

#### A-(3) CD-DST in human lung cancer cell lines

The cell viability of each lung cancer cell line following exposure to 0.2 μM of erlotinib for 7 days was 87.1 ± 0.56, 72.2 ± 5.20, 55.8 ± 8.1, and 0.63 ± 0.19% for H460, A549, H1975, and HCC827 cell lines, respectively (n = 3 each). HCC827 cell line showed significant growth inhibition following exposure to erlotinib (p = 0.028). On the other hand, no growth inhibitory effect was observed in wild-type EGFR cell lines with exposure to 0.2 μM erlotinib (Fig 1-e). The viability of each lung cancer cell line following exposure to 0.6 μM crizotinib for 7 days was 93.7 ± 3.13, 75.5 ± 7.43, and 20.1 ± 2.13% for H460, A549, and H3122 cell lines, respectively (n = 3 each), and H3122 showed significant cell growth inhibition following exposure to crizotinib (Fig 1-f).

### B. Clinical study

Total thirty-five (SDI: 23, CD-DST: 12) patients were enrolled into this study (Table 1). Four of 24 cases (16.7%) in men and seven of 11 cases (63.6%) in women expressed EGFR mutation. By histological subtype, 8 of 11 cases (72.7%) of papillary adenocarcinoma, 3 of 4 cases (75%) of BAC; bronchiolo-alveolar carcnoma expressed in, and one of 7 cases (14.3%) of acinar adenocarcinoma expressed EGFR mutation.

**Table 1:** Demography of patients enrolled into in-vitro drug sensitivity tests.

	EGFR	
	mutation positive	wild type
N	11(3)	24 (9)
gender		
Male	4 (1)	20 (6)
Female	7 (2)	4 (3)
Age	73.7±7.48	69.1 ±11.6
Histology		
Adeno.	11 (3)	14 (7)
Solid	0	1 (1)
Papillary	8 (2)	3 (2)
Acinar	1 (0)	6 (3)
Lepidic	0 (0)	2 (1)
BAC	2 (1)	1 (0)
Poorly differentiated	0 (0)	1 (0)
Squamous	0 (0)	7 (2)
Large	0 (0)	1 (0)
Adeno-squamous	0 (0)	2 (0)
EGFR mutation		
Exon 19 del E746-A750	6 (2)	N/A
Exon 21: L858R	3 (1)	N/A
Exon 21: L861Q	1 (0)	N/A
Exon 21: G719A	1 (0)	N/A

The number of patients enrolled either SDI or CD-DST. The numbers surrounded in a parenthesis showed the number for CDDST.

#### B-(1) SDI for clinical specimen

**Fig 2:**
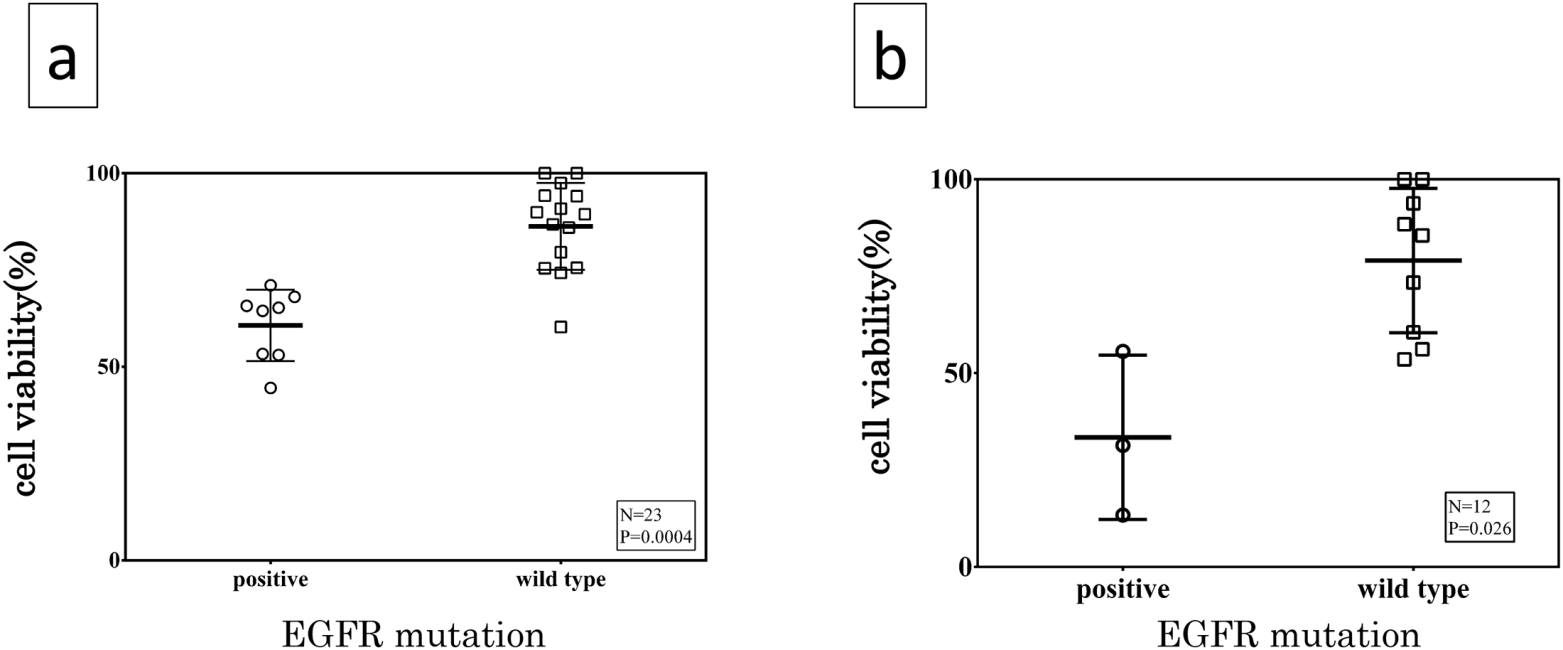
Growth inhibitory effect of erlotinib on surgically excised clinical lung cancer cells. Distribution map of cell viability evaluated in surgically resected fresh lung cancer tissue using (a) SDI following exposure to 20 μM erlotinib, with one case without *EGFR* mutation suppressed by 20 μM erlotinib and (b) CD-DST following exposure to 0.2 μM of erlotinib. Statistical significance observed in cell viability between two groups of *EGFR* mutation-positive and wild type (p = 0.026).

Evaluation of the growth inhibitory effect of erlotinib by the SDI method revealed that cancer cell viability was reduced concentration-dependently in the *EGFR* mutation-positive cases but hardly in the *EGFR*-negative cases even at high concentration of up to 20 mM (data not shown). In addition, following exposure to erlotinib 20 μM, the viability of *EGFR* wild-type cases was 86.3 ± 11.2% while that of the mutants was significantly lower at 60.0 ± 9.8% (p = 0.0004, Fig 2-a).

#### B-(2) CD-DST for clinical specimen

**Fig 3:**
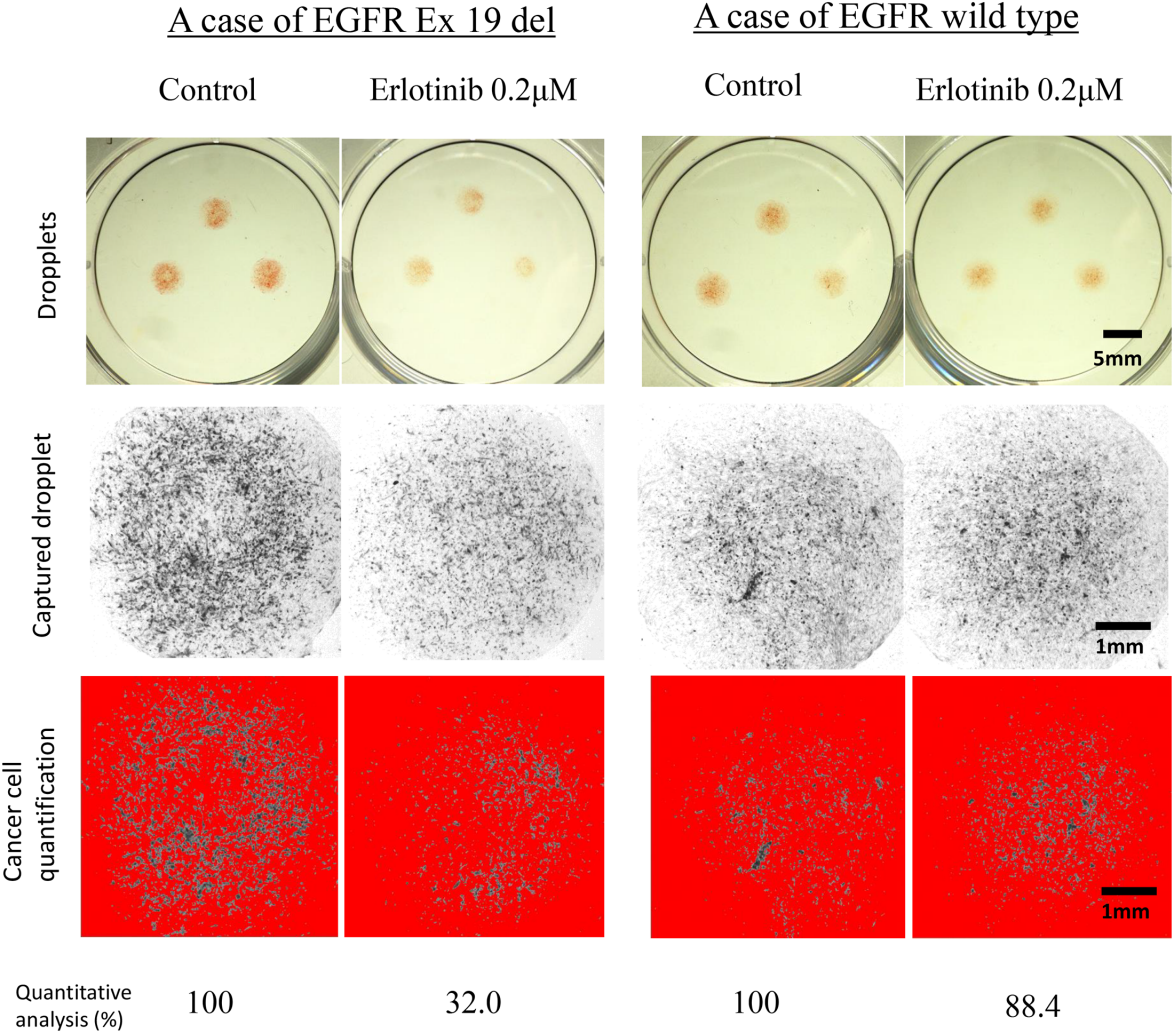
Two representative cases evaluated by CD-DST. CD-DST results of two representative cases, with cancer cells in each droplet cultured with or without 0.2 μM of erlotinib for 7 days. Top row shows photographs of collagen gel droplets containing cancer cells, middle row shows droplets scanned using dedicated image scanning system, and bottom row shows droplets with eliminated fibroblasts stained weaker than the cut-off point. Case with exon 19 del in *EGFR* (left two columns) shows number of cancer cells were reduced to 32.0% of untreated control when exposed to 0.2 μM erlotinib. Patient with *EGFR* wild-type case (right two columns) showed 88.4% growth compared to the control.

Following exposure to 0.2 μM erlotinib, the viability of the *EGFR* mutants and wild-types was 33.5 ± 21.2 and 79.0 ± 18.6%, respectively, and there was a significant difference in the cell viability (p = 0.026, Fig 2-b). The representative CD-DST results of both *EGFR* mutant and wild type cases are illustrated in Fig 3. The data of EGFR mutation status and cell viability of each clinical patients were shown in S1 Table.

**Fig 4:**
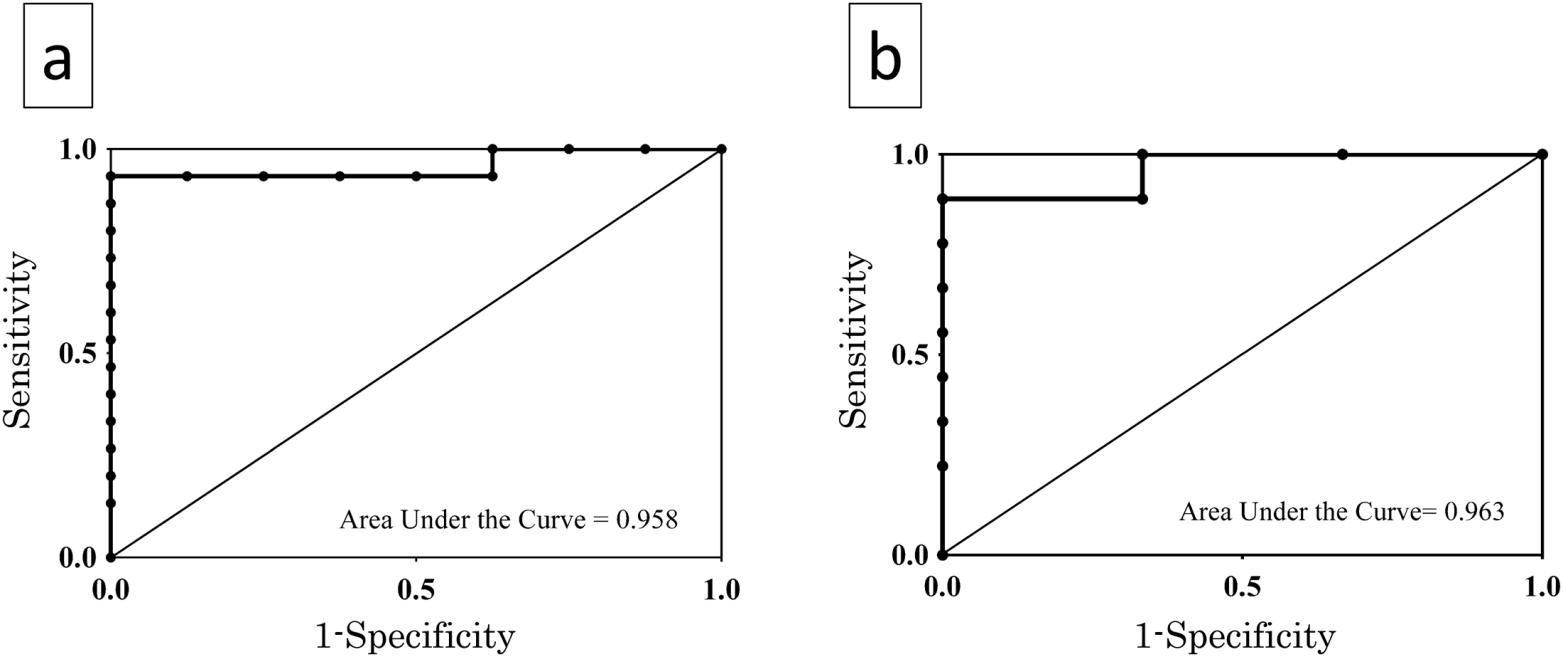
ROC curves. (a) ROC curves for cell viability evaluated using SDI in predicting *EFGR* mutation showing AUC of 0.958. (b) ROC curves for cell viability evaluated using collagen gel droplet embedded culture drug sensitivity test (CD-DST) in predicting *EGFR* mutation. AUC was 0.963.

The ROC curve was constructed to determine the cut-off value for predicting the presence of *EGFR* mutations from the growth inhibition rate of the in vitro drug sensitivity tests [24]. In the SDI test, the area under the curve (AUC) for cell viability was 0.958 (Fig 4-a). The ratio showed the best combination of sensitivity and specificity for the prediction of drug sensitivity at values > 72.7% (93.3 and 100% sensitivity and specificity, respectively). In the CD-DST, the ROC curve showed that AUC was 0.963 for cell viability (Fig 4-b) while the best combination of sensitivity and specificity for prediction of drug sensitivity was at 55.9% (88.9 and 100% sensitivity and specificity, respectively).

**Fig 5:**
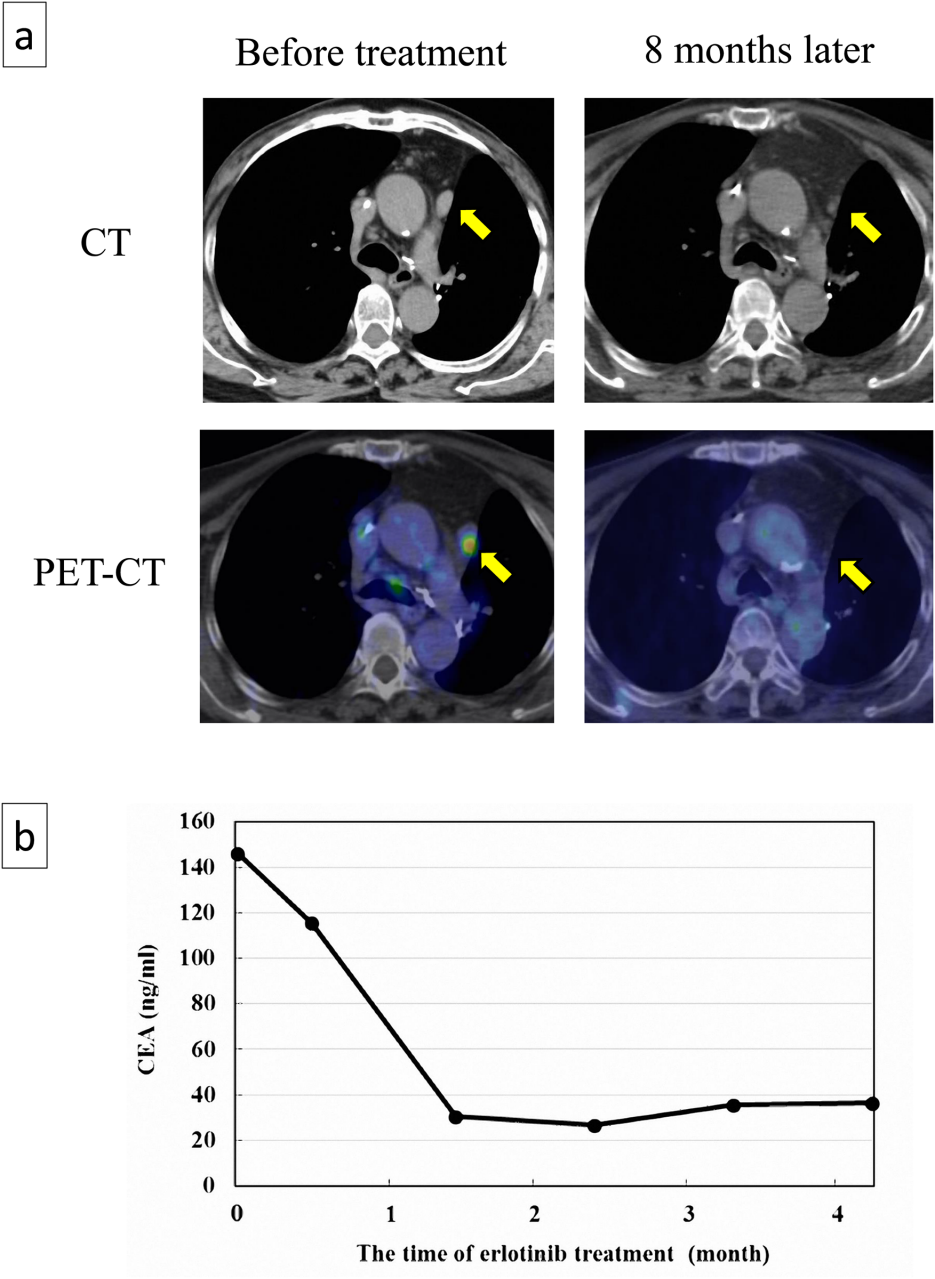
The two representative erlotinib-senstivive clinical cases treated with erlotinib. (a) Case #1 had exhibited 55.6% of cancer cell growth with erlotinib exposure in CD-DST. The cancer spread to the medianstinal lymph node (Yellow arrows). After erlotinib treatment, the mediastinal lymph node shrunk from 10.5 mm to 3.2 mm in size, and SUV of FDG decreased from 4.4 to 0.69. (b) Case #2 had exhibited 31.4% of cancer cell growth with erlotinib exposure in CD-DST, The patient caused the pleural dissemination with high level of CEA. Erlotinib treatment resulted in the reduction of CEA from 146.0 to 25.6 (ng/ml). 18F-FDG: 18F-fluorodeoxyglucose. PET: positoron emission tomography. SUV: standardized uptake value. CEA: Carciono-emboryonic antigen.

Prospectively, two patients with EGFR mutation got recurrent disease after the current study. The cell viabilities of the cancer cells derived from these patients were 55.6% and 31.4% with the exposure of erlotinib in CD-DST. Erlotinib exhibited the excellent clinical responses for these patients. The one patient had the enlarged metastatic lymph nodes. After 8 months of erlotinib treatment, the metastatic lymph node shrunk from 10.5 mm to 3.2mm and SUV (standardized uptake value) of 18F-FDG (18F-fluorodeoxyglucose) reduced from 4.44 to 0.69. The effect was judged as CR (complete response) radiologically (Fig 5-a). The other patient had the pleural dissemination with the elevation of CEA (Carcinoembryonic antigen); one of the serological tumor markers of lung cancer. As a result of Erlotinib treatment, the level of CEA reduced from 146.0 to 25.6 (ng/ml) (Fig 5-b). The effect of the molecular targeting drugs for patients with positive sensitivity in in-vitro drugsensitivity tests and negative gene alterlation is to be revealed.

## Discussion

In the current study, we first confirmed that erlotinib and crizotinib exhibited dose-dependent growth inhibition of cultured lung cancer cells. The inhibitory effect of erlotinib was stronger in lung cancer cell-lines with *EGFR* mutation than it was in those without the mutation. Similarly, crizotinib showed stronger inhibition of lung cancer cell lines with a recurrent gene fusion between *EML4* and *ALK* than it did in those without the fusion gene. Second, we demonstrated that the growth inhibitory effect of erlotinib evaluated by either the SDI or CD-DST was significantly correlated to the *EGFR* mutation status in the clinical study using the surgically resected lung cancer tissue specimens. Cell culture-based in vitro drug sensitivity tests may be able to predict the sensitivity of cancer cells to various molecular target drugs under development for future clinical application.

SDI performed on clinical samples showed growth inhibition in patients with *EGFR* mutation. However, SDI required higher concentrations of erlotinib for cell proliferation inhibition than are usually obtained in the blood following the administration of standard oral doses [25]. Furthermore, the SDI required more than six sets of 1.0 × 10^6^ cells, while CD-DST required less than 1.0 × 10^5^ cells to perform the examinations. With less amount of tissue requirement,CD-DST hardly affects the pathological examination.

In CD-DST, erlotinib showed growth inhibitory effect with lower concentration than that of MTS assay. HCC827 cell growth was suppressed almost completely by exposure to 0.01 μM erlotinib while that of H460, A549 and H1975 cells was slightly suppressed following exposure to 0.2 μM, after which it increased concentration-dependently manner. These results suggest that CD-DST detected the difference in the growth inhibitory effects in all cases at concentration of erlotinib as low as 0.2 μM, which is lower than serum concentrations of patients who were regularly administered 150 mg of erlotinib daily [25].

One controversial result was reported 0.35 μM of gefitinib failed to inhibit tumor growth in patients who are mutation-positive in a previous study that investigated the correlation of *EGFR* mutation and drug sensitivity using the CD-DST [21]. Our study used the PCM4 serum-free culture medium with reduced growth factors instead of the conventional PCM2, this maybe the reason why our result exhibit correlation EGFR mutation with their sensitivity successfully. But the composition of PCM4 is not released because of protected patent.

The histoculture drug response assay (HDRA), is another drug sensitivity test that uses a three dimensional collagen gel, and the dose-response curve of gefitinib for lung cancer was reported using this method. However, the investigation did not include correlating the *EGFR* mutation and effects of gefitinib [26].

In the future, new molecular abnormalities may become evident along with the expected development of relevant molecular targeted drugs for their treatment. Therefore, there is an urgent requirement to develop methods that can simultaneously predict the clinical responses of each molecular targeted drug at a reasonable cost. Recent advances in genetic search techniques have enabled the reporting of comprehensive gene expression profiling systems [27, 28]. However, these systems are still uncommon. Furthermore, for the prediction of sensitivity to ALK inhibitors, *EML4-ALK* should be detected by FISH or IHC rather than gene mutation analysis [29, 30]. It is complicated to perform the various kinds of screening required to detect gene alterations. Cell-culture based in-vitro growth assays have advantage because they examine drugs at simultaneously. The CD-DST in particular, has the advantage of being minimizing the volume of cancer tissue (3mm cubed) in the evaluation of clinical tissue sample, compared to the SDI (10mm cubed).

Study limitations: In this study, we demonstrated that the in vitro drug sensitivity was significantly correlated to *EGFR* mutation status. However, both *EGFR* mutation analysis and in vitro drug sensitivity tests are the potential predicting factors of clinical responses to EGFR-TKI therapy. Therefore, the validity of the predicting factors should ultimately be assessed by investigating their correlation to clinical responses, which requires clarification in future prognosis-related investigations.

## Supporting Information

S1 Table Characteristics of clinical patients. (PDF)
