# Correction: *In Vitro* Drug Sensitivity Tests to Predict Molecular Target Drug Responses in Surgically Resected Lung Cancer

**DOI:** 10.1371/journal.pone.0166505

**Published:** 2016-11-08

**Authors:** Ryohei Miyazaki, Takashi Anayama, Kentaro Hirohashi, Hironobu Okada, Motohiko Kume, Kazumasa Orihashi

There is an error in the third sentence of the seventh paragraph of the Results. The correct sentence is: The ratio showed the best combination of sensitivity and specificity for the prediction of drug sensitivity at values > 72.7% (100 and 93.3% sensitivity and specificity, respectively).

There is an error in the last sentence of the seventh paragraph of the Results. The correct sentence is: In the CD-DST, the ROC curve showed that AUC was 0.963 for cell viability (Fig 4b) while the best combination of sensitivity and specificity for prediction of drug sensitivity was at 55.9% (100 and 88.9% sensitivity and specificity, respectively).

There is an error in Fig 4. Please see the corrected Fig 4 here.

**Fig 4:**
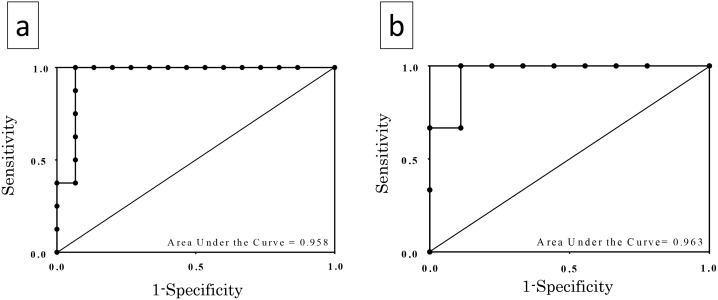
ROC curves. (a) ROC curves for cell viability evaluated using SDI in predicting *EFGR* mutation showing AUC of 0.958. (b) ROC curves for cell viability evaluated using collagen gel droplet embedded culture drug sensitivity test (CD-DST) in predicting *EGFR* mutation. AUC was 0.963.
