# Supplementary material for: In Vitro Drug Sensitivity Tests to Predict Molecular Target Drug Responses in Surgically Resected Lung Cancer
Source: PLoS One. 2016 Apr 12;11(4):e0152665. doi: 10.1371/journal.pone.0152665 (PMC4829246; doi:10.1371/journal.pone.0152665)
Supplement: S1 Table — (PDF) [file pone.0152665.s001.pdf]

Pt. No	age	gender	histology	subtype	GEFR mutation	cell viability
1	73	M	Adeno	Papillary	Ex19 L746-E750	65.3
2	65	M	Adeno-squamous		–	100
3	80	M	Squamous		–	100
4	67	M	Adeno	Papillary	G719A	65.7
5	83	F	Adeno	BAC	L858R	44.6
6	83	F	Adeno	Papillary	L858R	68.1
7	35	M	Adeno	Lepidic	–	89.9
8	64	M	Squamous		–	89.4
9	69	F	Adeno	BAC	Ex19 L746-E750	71.1
10	77	F	Adeno	Papillary	L861Q	53
11	77	F	Adeno	Acinar	Ex19 L746-E750	53.3
12	78	F	Adeno	Papillary	–	86
13	82	M	Large		–	74.2
14	61	M	Adeno	Poorly-differentiated	–	75.6
15	70	M	Adeno	Papillary	–	79.6
16	56	M	Adeno	Acinar	–	60.3
17	81	M	Adeno	Acinar	–	94.2
18	64	M	Adeno	Papillary	Ex19 L746-E750	64.4
19	83	M	Adeno-squamous		–	90.8
20	69	M	Squamous		–	94.1
21	81	M	Adeno	Acinar	–	86.8
22	67	M	Squamous		–	75.5
23	49	M	Squamous		–	97.5
24	69	F	Adeno	Papillary	Ex19 L746-E750	55.6
25	63	F	Adeno	Acinar	–	100
26	84	F	Adeno	Papillary	Ex19 L746-E750	32
27	66	M	Adeno	Solid	–	60.5
28	81	F	Adeno	Acinar	–	88.4
29	76	F	Adeno	Acinar	–	73.4
30	64	M	Squamous		–	53.5
31	70	M	Adeno	Papillary	–	100
32	65	M	Adeno	BAC	L858R	13.4
33	80	M	Squamous		–	93.8
34	65	M	Adeno	Lepidic	–	85.5
35	72	M	Adeno	Papillary	–	56.1
